# Guideline-Concordant Surgical Care for Lobular Versus Ductal Inflammatory Breast Cancer

**DOI:** 10.1245/s10434-024-15540-1

**Published:** 2024-06-17

**Authors:** Yoshiko Iwai, Stephany Perez-Rojas, Samantha M. Thomas, Audree B. Tadros, Steven G. Woodward, Jennifer Q. Zhang, Leisha C. Elmore, Gary M. Freedman, Julia C. Tchou, Aaron D. Bleznak, Oluwadamilola M. Fayanju

**Affiliations:** 1 University of North Carolina School of Medicine, Chapel Hill, NC USA; 2 Division of Breast Surgery, Department of Surgery, Perelman School of Medicine, The University of Pennsylvania, Philadelphia, PA USA; 3 Department of Biostatistics and Bioinformatics, Duke University School of Medicine, Durham, NC USA; 4 Duke Cancer Institute, Duke University School of Medicine, Durham, NC USA; 5 Breast Service, Department of Surgery, Memorial Sloan Kettering Cancer Center, New York, NY USA; 6 Rena Rowan Breast Center, Abramson Cancer Center, Penn Medicine, Philadelphia, PA USA; 7 Department of Radiation Oncology, Perelman School of Medicine, The University of Pennsylvania, Philadelphia, PA USA; 8 Leonard Davis Institute of Health Economics (LDI), The University of Pennsylvania, Philadelphia, PA USA; 9 Ann B. Barshinger Cancer Institute, Penn Medicine Lancaster General Health, Lancaster, PA USA; 10 Penn Center for Cancer Care Innovation, Abramson Cancer Center, Philadelphia, PA USA; 11 Present Address: Luminis Health Anne Arundel Medical Center, Annapolis, MD USA; 12 Present Address: Riverside Regional Medical Center, Newport News, VA USA

## Abstract

**Introduction:**

Quality of surgical care is understudied for lobular inflammatory breast cancer (IBC), which is less common, more chemotherapy-resistant, and more mammographically occult than ductal IBC. We compared guideline-concordant surgery (modified radical mastectomy [MRM] without immediate reconstruction following chemotherapy) for lobular versus ductal IBC.

**Methods:**

Female individuals with cT4dM0 lobular and ductal IBC were identified in the National Cancer Database (NCDB) from 2010–2019. Modified radical mastectomy receipt was identified via codes for “modified radical mastectomy” or “mastectomy” and “≥10 lymph nodes removed” (proxy for axillary lymph node dissection). Descriptive statistics, chi-square tests, and *t*-tests were used.

**Results:**

A total of 1456 lobular and 10,445 ductal IBC patients were identified; 599 (41.1%) with lobular and 4859 (46.5%) with ductal IBC underwent MRMs (*p* = 0.001). Patients with lobular IBC included a higher proportion of individuals with cN0 disease (20.5% lobular vs. 13.7% ductal) and no lymph nodes examined at surgery (31.2% vs. 24.5%) but were less likely to be node-negative at surgery (12.7% vs. 17.1%, all *p* < 0.001). Among those who had lymph nodes removed at surgery, patients with lobular IBC also had fewer lymph nodes excised versus patients with ductal IBC (median [interquartile range], 7 (0–15) vs. 9 (0–17), *p* = 0.001).

**Conclusions:**

Lobular IBC patients were more likely to present with node-negative disease and less likely to be node-negative at surgery, despite having fewer, and more frequently no, lymph nodes examined versus ductal IBC patients. Future studies should investigate whether these treatment disparities are because of surgical approach, pathologic assessment, and/or data quality as captured in the NCDB.

**Supplementary Information:**

The online version contains supplementary material available at 10.1245/s10434-024-15540-1.

Inflammatory breast cancer (IBC) is rare, constituting only 1–5% of breast cancers, yet contributes to approximately 10% of breast cancer-related mortality in the United States.^1,2^ Treatment for IBC has improved over recent decades with increasingly effective systemic therapy, particularly with regards to targeted therapies (e.g., trastuzumab, pertuzumab) for HER2-positive (HER2+) disease.^3-5^

Current guideline-concordant care (GCC) for IBC dictates a course of treatment that typically begins with neoadjuvant chemotherapy (NACT) followed by modified radical mastectomy (MRM) without immediate reconstruction; postmastectomy radiation therapy (PMRT); and appropriate adjuvant therapy by tumor subtype (e.g., endocrine therapy for hormone receptor-positive [HR+] disease) and response to NACT.^6^ Inflammatory breast cancer is a clinical diagnosis and can manifest as any histology but most commonly presents as ductal histology in nearly 90% of cases.^5,7,8^ Thus, most literature and treatment recommendations are based on cumulative experience with ductal IBC. Furthermore, receipt of guideline-concordant surgery for lobular IBC is relatively understudied. We compare receipt of guideline-concordant surgery (i.e., MRM without immediate reconstruction after chemotherapy) for lobular versus ductal IBC.

## Methods

Female patients diagnosed with cT4dM0 ductal or lobular breast cancer between 2010 and 2019 from the National Cancer Database (NCDB) were identified. Patients missing survival status or with metastatic disease (M1) were excluded.

Guideline-concordant surgery was defined as modified radical mastectomy (MRM) without immediate reconstruction following neoadjuvant chemotherapy. We defined MRM as any of the following: total (simple) mastectomy with ≥10 lymph nodes removed, total simple mastectomy with ≥10 lymph nodes removed without removal of uninvolved contralateral breast, total simple mastectomy with ≥10 lymph nodes removed with removal of uninvolved contralateral breast, MRM, MRM without removal of uninvolved contralateral breast, MRM with removal of uninvolved contralateral breast, and radical mastectomy, NOS. The specific codes that were used to identify potentially guideline-concordant surgeries are summarized in Appendix 1 and Appendix 2. A sizable proportion of patients with primary surgery site codes for MRM were documented as having no lymph nodes removed despite axillary lymph node dissection (ALND) being a standard component of MRMs. For most analyses, we limited our cohort of guideline-concordant surgery recipients to those who had ≥10 lymph nodes removed and examined, given common use of this criterion to define ALND (see *Limitations*). However, we also performed sensitivity analyses in which we did not exclude MRM recipients with <10 lymph nodes from being categorized as having received guideline-concordant surgery to account for potential miscoding of ALND and under-capture of lymph node yield.

Demographic factors for patients who met inclusion criteria were abstracted. Patient characteristics were summarized with N (%) for categorical variables and median (interquartile range [IQR]) for continuous variables. Chi-square tests and *t*-tests were used to test for differences in categorical and continuous variables, respectively. Effective sample sizes are included for all tables and figures. No adjustments were made for multiple comparisons. *P* < 0.05 was deemed significant for all analyses, which were conducted using SAS version 9.4 and R version 4.2.2.

This study operated under a waiver of HIPAA after being reviewed by the University of Pennsylvania Institutional Review Board (Protocol #831190). NCDB guidelines were followed to protect patient privacy, and counts < 20 were not reported. The study followed the Strengthening the Reporting of Observational Studies in Epidemiology (STROBE) Statement guidelines for reporting observational studies.^9^

## Results

**Fig. 1:**
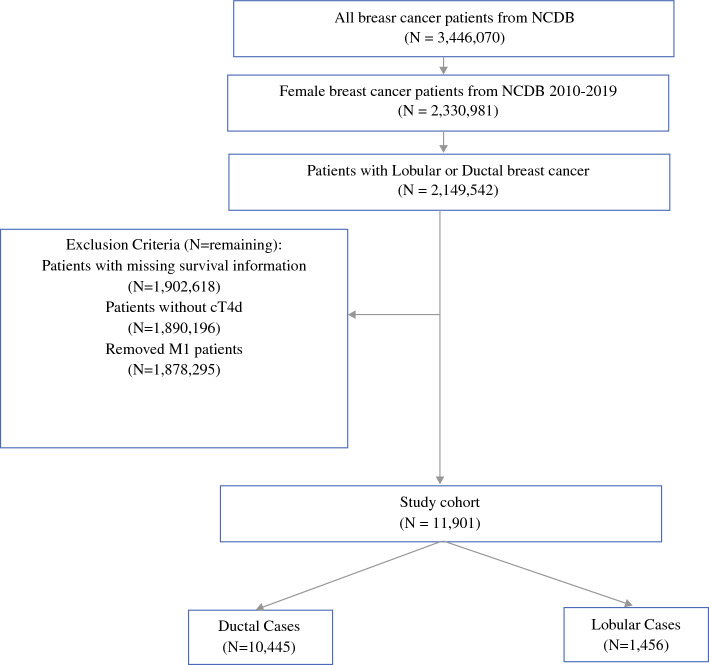
Consort diagram for analytical cohort using the national cancer database, 2010–2019. *NCDB* National Cancer Database

Of the 3,446,070 breast patients identified in the NCDB from 2010–2019, only 11,901 female patients with nonmetastatic ductal (n = 10,445) or lobular (n = 1,456) IBC were included in our analytic cohort (Fig. 1).

Patient characteristics are summarized in Table 1. Patients with lobular IBC were older compared with patients with ductal IBC (56.7% lobular vs. 43.9% ductal ≥ 60 years; *p* < 0.001); 75.3% of patients with lobular IBC and 68.7% of patients with ductal IBC were non-Hispanic (NH) white, and 15.1% of patients with lobular IBC and 10.4% of patients with ductal IBC were NH black. Biomarker subtypes were less evenly distributed within the lobular cohort, which had significantly higher rates of HR+/HER2− disease (55.3% lobular vs. 34.9% ductal) and lower rates of HER2+ disease (13.4% lobular vs. 24.6% ductal) and TNBC (13.4% lobular vs. 24.6% ductal, *p* < 0.001). Patients with lobular IBC also were more likely to have tumors >5 cm at the time of diagnosis (68.3% lobular vs. 61.4% ductal, *p* < 0.001).

**Table 1:** Patients with ductal and lobular inflammatory breast cancer in the National Cancer Database, 2010–2019

		Ductal	Lobular	All patients	*p*
		*n* = 10,445	*n* = 1456	*N* = 11,901	
Age median (IQR)		57 (48–66)	62 (52–71)	58 (49–67)	< 0.001
Age	<50	2835 (27.1)	294 (20.2)	3129 (26.3%)	< 0.001
	50–59	3,019 (28.9)	336 (23.1)	3,355 (28.2%)	
	60–69	2,573 (24.6)	415 (28.5)	2,988 (25,1%)	
	70–79	1,265 (12.1)	240 (16.5)	1,505 (12.6%)	
	>80	753 (7.2)	171 (11.7)	924 (7.8%)	
Race/ethnicity	AI/AN	43 (0.4)	10 (0.7)	53 (0.4%)	< 0.001
	Hispanic	741 (7.1)	79 (5.4)	820 (6.9%)	
	NH Asian/PI	305 (2.9)	25 (1.7)	330 (2.8%)	
	NH black	2031 (10.4)	220 (15.1)	2,251 (18.9%)	
	NH white	7177 (68.7)	1097 (75.3)	8274 (69.5%)	
	Unknown	148 (1.4)	25 (1.7)	173 (1.5%)	
Subtype	HER2+	3604 (34.5)	369 (25.3)	3973 (33.4%)	< 0.001
	HR+/HER2−	3646 (34.9)	805 (55.3)	4451 (37.4%)	
	TNBC	2566 (24.6)	195 (13.4)	2761 (23.2%)	
	Unknown	629 (6.0)	87 (6.0)	716 (6.0%)	
Tumor size (cm)	<1	346 (3.3)	36 (2.5)	382 (3.2%)	< 0.001
	1–1.9	860 (8.2)	115 (7.9)	975 (8.2%)	
	2–2.9	998 (9.6)	97 (6.7)	1,095 (9.2%)	
	3–3.9	956 (9.2)	127 (8.7)	1083 (9.1%)	
	4–4.9	873 (8.4)	86 (5.9)	959 (8.1%)	
	>5	6,412 (61.4)	995 (68.3)	7407 (62.2%)	
Node-negative	No**	5895 (56.4)	798 (54.8)	6693 (56.2%)	< 0.001
	Yes**	1848 (17.7)	185 (12.7)	2033 (17.1%)	
	Nodes not examined	2556 (24.5)	454 (31.2)	3010 (25.3%)	
	Unknown	146 (1.4)	19 (1.3)	165 (1.4%)	
Lymph node count	No nodes examined	2556 (24.5)	454 (31.2)	3010 (25.3%)	< 0.001
	<10	2,248 (21.5)	310 (21.3)	2,558 (21.5%)	
	≥10	3711 (35.5)	455 (31.3)	4,166 (35%)	
	Unknown	1930 (18.5)	237 (16.3)	2167 (18.2%)	
Types of breast surgery	Modified radical mastectomy	4230 (40.5%)	519 (35.6%)	4749 (39.9%)	0.002
	Total mastectomy	1768 (16.9%)	244 (16.8%)	2012 (16.9%)	
	Other surgery	1,280 (12.3%)	192 (13.2%)	1,472 (12.4%)	
	None	3167 (30.3%)	501 (34.4%)	3668 (30.8%)	
Guideline-concordant surgery (GCS) vs. other types of surgery	GCS (i.e., MRM and/or TM with ≥10 LN excised)	4859 (46.5%)	599 (41.1%)	5458 (45.9%)	< 0.001
	Other surgery	2,419 (23.2%)	356 (24.5%)	2775 (23.3%)	
	None	3167 (30.3%)	501 (34.4%)	3668 (30.8%)	
Clinical N classification	cN0	1430 (13.7%)	298 (20.5%)	1728 (14.5%)	< 0.001
	cN1	5230 (50.1%)	684 (47.0%)	5914 (49.7%)	
	cN2	1600 (15.3%)	200 (13.7%)	1800 (15.1%)	
	cN3	1903 (18.2%)	226 (15.5%)	2129 (17.9%)	
	Unknown	282 (2.7%)	48 (3.3%)	330 (2.8%)	
Pathological T classification	pT0	1404 (13.50%)	136 (9.3%)	1540 (12.9%)	< 0.001
	pT1	943 (9.0%)	95 (6.5%)	1,038 (8.7%)	
	pT2	796 (7.6%)	116 (8.0%)	912 (7.7%)	
	pT3	595 (5.7%)	178 (12.2%)	773 (6.5%)	
	pT4	1702 (16.3%)	232 (15.9%)	1934 (16.3%)	
	Other	5005 (47.9%)	699 (48.0%)	5704 (47.9%)	
Pathological N classification	pN0	2547 (24.4%)	272 (18.7%)	2819 (23.7%)	< 0.001
	pN1	1684 (16.1%)	195 (13.4%)	1879 (15.8%)	
	pN2	1,303 (12.5%)	218 (15.0%)	1,521 (12.8%)	
	pN3	827 (7.9%)	151 (10.4%)	978 (8.2%)	
	Other	4084 (39.1%)	620 (42.6%)	4704 (39.5%)	
Received radiation	Administered	3228 (31.0%)	478 (32.8%)	3716 (31.2%)	0.334
	None	5669 (54.3%)	777 (53.4%)	6446 (54.2%)	
	Possible inappropriate administration (e.g., whole breast)	789 (7.6%)	113 (7.8%)	902 (7.6%)	
	Other	749 (7.25%)	88 (6%)	837 (7%)	
Received endocrine therapy*					< 0.001
	Yes	2786 (76.4%)	655 (81.4%)	3441 (77.3%)	
	No	860 (23.6%)	150 (18.6%)	1010 (22.7%)	

^*^HR + patients only; N = 4,451

^**^Lymph nodes removed at surgery and examined by pathologist.

*IQR* Interquartile range; *AI/AN* American Indian/Alaska Native; *NH* Non-Hispanic; *TNBC* Triple-negative breast cancer; *MRM* Modified radical mastectomy

Among patients undergoing axillary surgery, higher proportion of patients with lobular IBC presented with cN0 disease (20.5% lobular vs. 13.7% ductal), whereas a lower proportion were node-negative at surgery compared with ductal IBC patients (12.7% lobular vs. 17.1% ductal; both *p* < 0.001). Furthermore, more patients with lobular IBC had no nodes examined from surgery compared with patients with ductal IBC (31.2% lobular vs. 24.5% ductal, *p* < 0.001). Of these 2556 patients, 645 had breast surgery and no nodes examined: 155 were coded as having technically undergone MRM, and 294 underwent total mastectomy. Fewer patients with lobular IBC underwent MRM (35.6% lobular vs. 40.5% ductal), and a higher proportion of patients with lobular IBC had no surgery compared with patients with ductal IBC (34.4% lobular vs. 30.3% ductal; *p* = 0.002). Overall, 19.5% of patients with lobular IBC and 20.2% of patients with ductal IBC received all components of GCC (i.e., neoadjuvant chemotherapy, MRM without immediate reconstruction, postmastectomy radiation therapy, and if HR+, endocrine therapy).

The distribution of patients undergoing MRM by lymph node count is summarized in Table 2. Fewer patients with lobular IBC had ≥10 nodes examined compared with patients with ductal disease (31.3% lobular vs. 35.5% ductal, *p* < 0.001), but the proportion of patients with one to nine nodes retrieved (21.3% lobular vs. 21.5% ductal) or with an unknown number of lymph nodes retrieved (16.3% lobular vs. 18.5% ductal) were similar between the two groups. The median number of lymph nodes excised was lower among patients with lobular IBC compared with ductal IBC (median (interquartile range [IQR]), 7 (0−15) lobular vs. 9 (0−17) ductal, *p* = 0.001). Overall, fewer patients with lobular IBC received guideline-concordant surgery, i.e., MRM as defined by coding or definition of total mastectomies with ≥10 nodes removed (41.1% lobular vs. 46.5% ductal, *p* < 0.001); this finding remained true in sensitivity analyses in which we did not apply lymph node criteria restrictions (Table 2).

**Table 2:** Modified radical mastectomies and variations based on lymph node count restriction in the National Cancer Database, 2010–2019

		Ductal	Lobular	*p*
		*n* = 10,445 (%)	*n* = 1,456 (%)	
Lymph nodes excised count	No LN examined	2556 (24.5%)	454 (31.2%)	< 0.001
	1–9 LN examined	2248 (21.5%)	310 (21.3%)	
	≥10 LN examined	3711 (35.5%)	455 (31.3%)	
	Unknown	1930 (18.5%)	237 (16.3%)	
Median no. lymph nodes excised (IQR)		9 (0–17)	7 (0–15)	0.001
Surgery types	Modified radical mastectomy	4,230 (40.5%)	519 (35.6%)	0.002
	Total mastectomy	1768 (16.9%)	244 (16.8%)	
	Other surgery	1280 (12.3%)	192 (13.2%)	
	None	3167 (30.3%)	501 (34.4%)	
Inclusive surgery types	MRM or TM with ≥ 10 LN excised	4859 (46.5%)	599 (41.1%)	< 0.001
	Other surgery	2419 (23.2%)	356 (24.5%)	
	None	3167 (30.3%)	501 (34.4%)	

*IQR* Interquartile range; *LN* Lymph node; *MRM* Modified radical mastectomy; *TM* Total mastectomy

## Discussion

In this study of patients with nonmetastatic IBC, we found that more patients with lobular IBC presented with clinically node-negative disease, but fewer of these patients were node-negative at surgery, despite higher rates of omitted axillary surgery compared with patients with ductal IBC. This is the first study to our knowledge that compares guideline-concordant surgical management for lobular and ductal IBC.

Our findings suggest there are differences in the standardization of surgical treatment among patients with different subtypes of IBC. It is unclear whether these differences are due to disparate surgical approaches, variable preoperative and/or pathologic assessment, or differences in data quality as captured at institutions contributing to the NCDB. Lobular histology is frequently understaged on conventional preoperative imaging (i.e., mammograms and ultrasounds). Thus, some patients with lobular IBC may in fact have node-positive disease that was not detected due to not receiving diagnostic breast MRI.^10-13^ These findings suggest a potential role for alternative staging methods that may be more sensitive to lobular disease, such as ^18^F-fluoroestradiol (FES) PET, for patients who are HR+.^14^

The lower rate of guideline-concordant MRM and lower lymph node retrieval rates among patients with lobular IBC may be related to chemotherapy response, which can decrease lymph node yield; however, it is still unclear how this may differentially impact ductal versus lobular histologic subtypes. While literature on surgical approaches to IBC is limited, more aggressive surgical management may be associated with lower locoregional recurrence and more extensive axillary surgery with improved survival in node-positive patients.^15-17^ Our findings suggest the need for more accurate preoperative staging to ensure that patients with lobular IBC are receiving the appropriate surgery to maximize their chances of survival, particularly as surgical de-escalation of the axilla accelerates, despite the absence of evidence that it is safe in IBC.^15,18,19^ The high proportion of patients with lobular IBC who have no nodes examined in surgery is concerning for a potentially vital missed treatment opportunity and may represent poor adherence to current guidelines. Furthermore, inadequate surgery of the axilla can result in false reassurance of the degree of responsiveness to neoadjuvant chemotherapy. Accurate surgical staging is critical for therapeutic planning, especially in considering the role of adjuvant therapies for what is often chemotherapy-resistant disease.^15^

The differences we appreciated in lobular versus ductal IBC from patient demographics to clinical characteristics suggests a need to delineate IBC subtypes in research and treatment. Given the generally low prevalence of lobular IBC compared with ductal IBC, and the relatively low incidence of IBC within breast cancer pathology, lobular-specific guidelines are limited. We hypothesize that the surgical disparities in lobular IBC may be partially attributable to operative challenges in identifying, retrieving, and pathologically assessing the recommended number of nodes in addition to challenges with determining operative candidacy for MRM to begin with. Surgeon intent is not captured in the NCDB; thus, it also is difficult to assess how many operations were intended to be performed with higher lymph node yield. It is impossible to know whether omission of axillary surgery was because of on-treatment disease progression that prompted a change from planned MRM to planned palliative mastectomy. Future studies should seek to delineate histologic IBC types and future efforts should be devoted to developing treatment guidelines optimized for specific tumor biology and patient characteristics. Additional studies using multicenter data where surgeon intent can be captured may help to understand how much these treatment disparities are because of operative challenges and histology-specific difficulties with pathologic assessment.

Our findings highlight the importance of detailed and accurate data reporting for surgical procedures. The NCDB was used for this analysis due to its historically robust oncologic data.^20,21^ Yet, as documented by Rubenstein et al.’s comparison of national databases for breast surgical oncology, the NCDB uses a unique coding system which has potential for coding errors, as seen with lymph node coding prior to 2012.^22,23^ This is unlike the data included in databases such as the National Surgical Quality Improvement Program (NSQIP), where multiple Current Procedural Terminology (CPT) codes can be assigned to indicate surgery type, outcomes, and indications for the primary procedure; however, NSQIP does not capture cancer-related data as robustly as the NCDB.^22^ Assessing surgical standards of care for lobular versus ductal IBC using an additional database, such as the Surveillance, Epidemiology, and End Results (SEER) Program, may clarify the surgical differences appreciated in our analysis.

### Limitations

Limitations of our study include those that are inherent to retrospective studies using the NCDB, including selection bias and incomplete data reporting. Inflammatory breast cancer is a clinical diagnosis that can be difficult to diagnose and distinguish from locally advanced breast non-IBC, and this diagnostic challenge may have impacted the IBC data quality in the NCDB.^24^ Nevertheless, the treatment for both types of disease is similar, suggesting that the rates of guideline-concordant surgery that we observed likely are not significantly affected by any diagnostic misclassification that may have occurred. Similarly, the NCDB does not have a mechanism for central pathology review, necessitating reliance on hospital-level clinical data which may result in inaccurate categorization (e.g., locally advanced vs. noninflammatory breast cancer) and inability to confirm histology. We made the assumption that ≥10 lymph nodes represented ALND and <10 lymph nodes represented sentinel lymph node biopsy; yet, we recognize that there are potential coding errors in the NCDB, acknowledge that our selected categories do not reflect surgical intent, and acknowledge these categories may be impacted by response to NACT.^25,26^ To mitigate these potential errors, we conducted sensitivity analyses with and without lymph node count restrictions among patients who were coded as having received MRMs.

Finally, one of the reasons for nonreceipt of surgery may be progression to or delayed recognition of metastatic disease during neoadjuvant treatment, which may be more common with lobular IBC, prompting the conduct of palliative mastectomy with intentional omission of axillary surgery. Thus, in large part to optimize surgical care, we need to optimize systemic therapy for what is often chemotherapy-resistant disease. Indeed, our study highlights the persistent challenge of durable clinical response to systemic therapy for HR+ disease, which makes up a majority of lobular IBC and non-IBC. Fortunately, even in the time elapsed since the end of our study’s inclusion period, there have been significant advances in systemic therapy for patients with HR+ disease, including increased use of CDK4/6 inhibitors and emerging therapies from clinical trials, such as I-SPY.^27,28^ We hope that neoadjuvant use of some of these treatments and concomitant improvement in response among patients with IBC may facilitate more receipt of guideline-concordant surgery.

## Conclusions

Patients with lobular IBC were more likely to present with node-negative disease and less likely to be node-negative at surgery despite having fewer, and more frequently no, lymph nodes examined compared with ductal IBC patients. Future studies should investigate whether these treatment disparities are the result of differences in surgical approach, preoperative staging, pathologic assessment, and/or data quality as captured in the NCDB. Such knowledge would inform development of potentially more tailored treatments for particular histological subtypes of IBC.

### Electronic supplementary material

Below is the link to the electronic supplementary material.

Supplementary file1 (DOCX 32 kb)
