# Supplementary material for: Guideline-Concordant Surgical Care for Lobular Versus Ductal Inflammatory Breast Cancer
Source: Ann Surg Oncol. 2024 Jun 17;31(9):5929–36. doi: 10.1245/s10434-024-15540-1 (PMC11300632; doi:10.1245/s10434-024-15540-1)
Supplement: Supplementary file 1 — Supplementary file1 (DOCX 32 kb) [file 10434_2024_15540_MOESM1_ESM.docx]

**Appendix 1. Codes for regional node variables in the National Cancer Database, 2010-2019**

| **Definition** | **Code** |
| --- | --- |
| No nodes were examined | 00 |
| 1-89 nodes were examined. (Code the exact number of regional lymph nodes examined) | 1-89 |
| 90 or more nodes examined | 90 |
| No regional nodes removed, but aspiration or core biopsy of regional nodes was performed | 95 |
| Regional lymph node removal was documented as sampling, and the number of nodes is unknown/not stated | 96 |
| Regional lymph node removal was documented as dissection, and the number of nodes is unknown/not stated | 97 |
| Regional lymph nodes surgically removed but number of lymph nodes unknown or not stated, and not documented as sampling or dissection; nodes were examined, but the number is unknown | 98 |
| Unknown if regional nodes examined. Not applicable or negative. Not stated in patient record. | 99 |

**Appendix 2. Codes for the Primary Site of Breast Surgical Procedures in the National Cancer Database 2010-2019**

| **Code** | | | | **Definition** |
| --- | --- | --- | --- | --- |
| **00** |  |  |  | None; no surgery of primary site |
| **19** |  |  |  | Local tumor destruction, NOS |
| **20** |  |  |  | Partial mastectomy, NOS; less than total mastectomy, NOS |
|  | **21** | | | Partial mastectomy WITH nipple resection |
|  | **22** | | | Lumpectomy or excisional biopsy |
|  | **23** | | | Re-excision of the biopsy site for gross or microscopic residual disease |
|  | **24** | | | Segmental mastectomy (including wedge resection, quadrantectomy, tylectomy) |
| **30** | | | | Subcutaneous mastectomy |
| **40** | | | | Total (simple) mastectomy |
|  | **41** | | | WITHOUT removal of uninvolved contralateral breast |
|  |  | **43** | | With reconstruction NOS |
|  |  |  | **44** | Tissue |
|  |  |  | **45** | Implant |
|  |  |  | **46** | Combined (Tissue and Implant) |
|  | **42** | | | WITH removal of uninvolved contralateral breast |
|  |  | **47** | | With reconstruction NOS |
|  |  |  | **48** | Tissue |
|  |  |  | **49** | Implant |
|  |  |  | **75** | Combined (Tissue and Implant) |
| **76** | | | | Bilateral mastectomy for a single tumor involving both breasts, as for bilateral inflammatory carcinoma. |
| **50** | | | | Modified radical mastectomy |
|  | **51** | | | WITHOUT removal of uninvolved contralateral breast |
|  |  | **53** | | Reconstruction, NOS |
|  |  |  | **54** | Tissue |
|  |  |  | **55** | Implant |
|  |  |  | **56** | Combined (Tissue and Implant) |
|  | **52** | | | WITH removal of uninvolved contralateral breast |
|  |  | **57** | | Reconstruction, NOS |
|  |  |  | **58** | Tissue |
|  |  |  | **59** | Implant |
|  |  |  | **63** | Combined (Tissue and Implant) |
| **60** | | | | Radical mastectomy, NOS |
|  | **61** | | | WITHOUT removal of uninvolved contralateral breast |
|  |  | **64** | | Reconstruction, NOS |
|  |  |  | **65** | Tissue |
|  |  |  | **66** | Implant |
|  |  |  | **67** | Combined (Tissue and Implant) |
|  | **62** | | | WITH removal of uninvolved contralateral breast |
|  |  | **68** | | Reconstruction, NOS |
|  |  |  | **69** | Tissue |
|  |  |  | **73** | Implant |
|  |  |  | **74** | Combined (Tissue and Implant) |
| **70** | | | | Extended radical mastectomy |
|  | **71** | | | WITHOUT removal of uninvolved contralateral breast |
|  | **72** | | | WITH removal of uninvolved contralateral breast |
| **80** | | | | Mastectomy, NOS |
| **90** | | | | Surgery, NOS |
| **99** | | | | Unknown if surgery Performed |

Abbreviations: NOS (not otherwise specified)
